# Lipid Nanoparticle and Liposome Reference Materials: Assessment of Size Homogeneity and Long-Term −70 °C and 4 °C Storage Stability

**DOI:** 10.1021/acs.langmuir.2c02657

**Published:** 2023-02-07

**Authors:** Zygmunt J. Jakubek, Sam Chen, Josh Zaifman, Yuen Yi C. Tam, Shan Zou

**Affiliations:** † Metrology Research Center, National Research Council Canada, Ottawa, Ontario K1A 0R6, Canada; ‡ Integrated Nanotherapeutics Inc., 205-4475 Wayburne Drive, Burnaby, British Columbia V5G 4X4, Canada

## Abstract

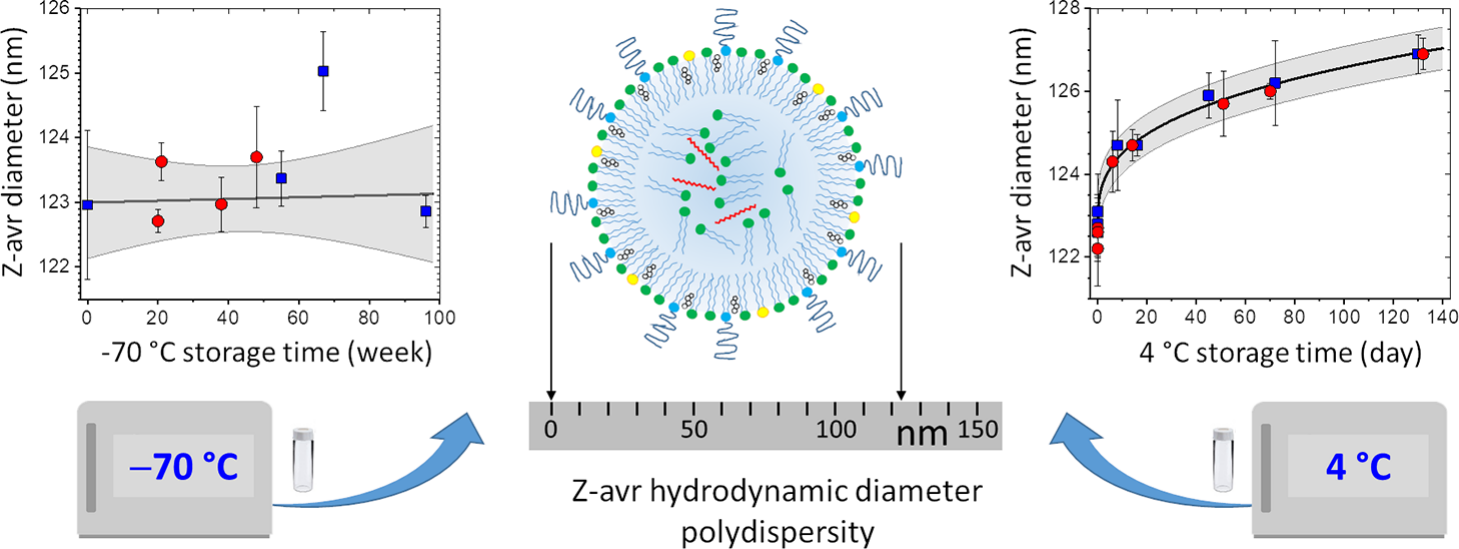

With recent advances and anticipated proliferation of lipid nanoparticle (LNP)-delivered vaccines and therapeutics, there is a need for the availability of internationally recognized reference materials of LNP systems. Accordingly, we developed six LNP and liposome (anionic, neutral, and cationic each) candidate reference material formulations and thoroughly characterized by dynamic light scattering their particle hydrodynamic size (Z-avr) and polydispersity. We also evaluated the particle size homogeneity and long-term −70 °C and 4 °C storage stability using multiple large sets of randomly selected vials for each formulation. The formulations stored at −70 °C remained stable and homogeneous for a minimum of 9 months. The Z-avr relative combined uncertainty and the long-term variability were both <1.3% for liposome formulations and anionic LNPs, (3.9% and 1.7%) for neutral LNPs, and (6.7% and 4.4%) for cationic LNPs. An inadvertent few-hour-long storage temperature increase to −35 °C due to a freezer malfunction resulted in a small change of the size and size distribution of anionic liposomes and LNPs but, unexpectedly, a larger size increase of the neutral and cationic liposomes (≤5%) and LNPs (≤25%). The mean Z-avr values of the LNPs stored at 4 °C appeared to slowly increase with *t*^1/3^, where *t* is the storage time, and the Z-avr between-vial heterogeneity and mean polydispersity index values appeared to decrease; no change was observed for liposomes. The size and size distribution evolution of LNPs stored at 4 °C was attributed to an incomplete equilibration of the formulations following the addition of sucrose prior to the initial freezing. Such a process of size increase and size distribution narrowing has not been previously discussed nor observed in the context of LNPs.

## Introduction

After several decades of lipid nanoparticle (LNP) research resulting in the development of a number of approved pharmaceuticals utilizing LNPs,^1^ LNP-based delivery systems took the center stage of the pharmaceutical industry with the introduction of the LNP-delivered nucleic acid-based therapeutics:^2^ first, the siRNA polyneuropathy drug^3^ and most recently and particularly mRNA covid-19 vaccines.^4^ With an anticipated proliferation of lipid-based delivery systems that are on the brink of revolutionizing medicine and escalation of related research and development, there is a need for robust relevant reference materials of key properties of such systems, in particular physicochemical properties of LNPs and liposomes. Such reference materials would aid methods development, facilitate inter laboratory comparison of physicochemical characteristics of novel delivery systems, simplify laboratory-to-laboratory translation of technologies, and ultimately further nanomedicine advances. While multiple companies develop internal reference or quality control materials of lipid-based drug delivery systems, there are currently no reference materials certified by national or international standardization bodies.

A reference material is defined by the ISO 17034 international standard as a “material, sufficiently homogeneous and stable with respect to one or more specified properties, which has been established to be fit for its intended use in a measurement process”.^5^ Among several physicochemical properties of the lipid-based nanocarriers, particle size and size distribution are critically important as they directly affect the encapsulated amount as well as delivery, distribution, release, and clearance of a therapeutic payload and, consequently, safety, potency, immunogenicity, and effectiveness of the system.^6−8^ Therefore, in the present report on our initial efforts to develop reference materials of lipid-based nanocarriers, we detail extensive characterization of particle size and size distribution of six LNP and liposome formulations, with particular emphasis on the stability and homogeneity of the formulations with respect to size.

Size and size distribution of LNPs and liposomes have been most frequently and most efficiently characterized by ensemble methods such as dynamic light scattering (DLS), static light scattering, and nanoparticle tracking analysis. Cryogenic transmission electron microscopy has also been an important size determination technique, but its true advantage is in aiding LNP morphology elucidation.^9^ Although compound multimodal methods such as multidetector asymmetric-flow field-flow fractionation),^10^ size-exclusion chromatography with multi-angle light scattering detection, or other fractionation-enhanced methods have been demonstrated to be advantageous for the determination of physicochemical attributes of lipid-based nanocarrier formulations, single technique methods are still routinely employed for physicochemical analyses.^11−13^ Therefore, DLS, a sizing technique commonly used in both single- and multimodal analyses, has primarily been utilized throughout this project.

The stability of LNP and liposome formulations is determined by the interplay of various molecular interactions and strongly depends on the composition of the dispersed particles and the dispersion medium as well as formulation processing and storage conditions. LNP-siRNA were shown to be the most stable without loss of efficacy when stored at 2 °C for over 150 days as compared to storage in the −20 °C freezer or at room temperatures.^14^ In contrast, LNP-mRNA were found to be long-term unstable in aqueous conditions, but when frozen in liquid nitrogen with the addition of 5% (w/v) sucrose or trehalose, they could remain stable for over 3 months.^15^ The recently developed mRNA-based covid-19 vaccines can be stored for a short term at (2 to 8) °C, but for long-term storage, (−80 to −60) °C (Pfizer-BioNTech) or −20 °C (Moderna) temperatures are required.^16^ Both vaccines are also stable at room temperatures, but only for several hours. While long-term storage stability is a highly desired property of LNP-based drug delivery systems, the best storage conditions and destabilization mechanisms remain not well understood. The storage temperatures of the drug delivery systems mentioned above should be indicative of storage temperatures required for therapeutically relevant LNP reference materials. Therefore, we extensively evaluated in this work the size stability and homogeneity of the LNP and liposome formulations stored for a long term at nominal temperatures of −70 °C and 4 °C. Results of the toxicity investigation have been reported in ref (17).

## Experimental Section

### Formulations

Six formulations, three each of LNP-siRNA and liposomes with varying surface charges and sizes, were produced at Integrated Nanotherapeutics Inc. (INT, Vancouver, BC, Canada) using INT proprietary lipids and scaffold technologies. The anionic, neutral, and cationic LNP formulations (ALNP, ζ = −26.1 mV; NLNP, ζ = 2.4 mV; and CLNP, ζ = 13.7 mV; respectively where ζ is a particle zeta potential) were produced by rapidly mixing the lipid components dissolved in ethanol with siRNA in an aqueous buffer at a volumetric flow rate ratio of 1:3 (ethanol to aqueous, combined flow rate 28 mL/min) at room temperature. The product was then dialyzed against 1x Dulbecco’s phosphate-buffered saline (dPBS) (Gibco, Thermo Fisher Scientific, Canada) at pH 7.4 for 24 h to remove residual ethanol and to raise the pH. The anionic, neutral, and cationic liposome formulations (AHC, ζ = −33.5 mV; NHC, ζ = −1.9 mV; and CHC, ζ = 6.2 mV; respectively) were produced by forcing appropriate lipid mixtures in 0.5x dPBS and 15% sucrose 12–15 times under a 300 PSI pressure through two-stacked 0.08 μm polycarbonate membranes using a Lipex liposome extruder heated to 65 °C. The LNP and liposome formulations were diluted with dPBS and aqueous sucrose solution to achieve the final lipid concentration in 0.5 dPBS/15% sucrose equal to 2 mg/mL except for NLNP that was 3 mg/mL. siRNA loaded into LNPs amounted to approximately 4.0 wt % of the total mass for ALNP and NLNP and 3.3 wt % for CLNP. Sets of 200 sequentially numbered vials, 1 mL/vial, were produced for each formulation; limited number of extra vials were also retained at INT for future reference. The vials were frozen in a −70 °C freezer. The boxed sets of 200 vials each were shipped overnight to NRC in thermally insulated styrofoam containers on dry ice. See also ref (17) for additional information about the formulations.

### Particle Size Characterization

Mean particle size and particle size distribution of the LNP and liposome formulations were evaluated by DLS using a Zetasizer Nano ZS (Malvern Instruments Ltd., Worcestershire, UK). Formulations were diluted 50-fold in 1x dPBS and measured at (25.0 ± 0.1) °C. Five repeat indications were acquired for each measurement, with each indication consisting of 21 runs 10 s long. The measurements were processed with the Zetasizer Software (ver. 7.11 or ver. 8.0, research grade; Malvern Panalytical, UK) using default values for all analysis parameters except for the viscosity (η = 0.9112 mPa·s) and refractive index (*n*_w_ = 1.334) of the medium. The Z-average (Z-avr) hydrodynamic diameter (sphere-equivalent scattered light intensity-weighted harmonic average hydrodynamic diameter) and polydispersity index (PdI) determined by cumulants analysis were the primary size and size distribution measurands used for the characterization of the six formulations.^18^

### Long-Term Storage Stability and Homogeneity Assessment

LNP and liposome candidate reference material formulations were stored in a freezer at temperatures ranging over (−80 to −70) °C and air-shipped between laboratories on dry ice. The storage stability and homogeneity of the formulations were monitored by periodically analyzing the size and size distribution (Z-avr and PdI) of the dispersed particles. Four sets of vials drawn by random stratified selection^19^ from each 200-unit batch of the six formulations were analyzed, three sets at NRC and one at INT. Table 1 summarizes the analyzes with the number of vials and aliquots measured and the approximate freezer storage time in weeks shown for each formulation and measurement set. Additional size and size distribution measurements were conducted on a small number of vials originally retained for reference and stored at INT at −70 °C. Upon completion of the measurements, the vials were transferred to a 4 °C refrigerator (3 °C to 5 °C normal temperature range) for long-term storage. The sets #1 and #2 stored at 4 °C were periodically removed from a refrigerator for the size and size distribution analysis.

**Table 1:** Summary of the −70 °C Storage Stability and Homogeneity Measurements on Four Sets of Vials per Formulation

	set #1	set #2	set #3	set #4
ALNP	12/3:3^a^	11/3:20	5/3:21	5/2:38
NLNP	10/3:3	10/3:22	5/3:22	9/2:39
CLNP	10/3:3	10/3:22	5/3:19	10/1:40
AHC	10/3:2	10/3:22	5/3:19	5/1:39
NHC	10/3:12	5/2:27	5/3:23	5/2:41
CHC	10/3:13	5/2:27	5/3:23	5/1:44

a Three numbers in each table cell listed as *x*/*y*:*z* are the number of vials analyzed (*x*), the number of aliquots per vial (*y*), and the approximate freezer storage time in weeks prior to the measurement (*z*).

### Additional Characterization

In addition to the −70 °C and 4 °C stability analyzes, short-term size stability at 37 °C was monitored as a part of the cytotoxicity study, and the results are presented elsewhere.^17^ Also, for selected representative vials, electrophoretic mobility and zeta potential were measured on formulations 10-fold diluted in water by mixed mode measurement phase analysis light scattering using a Zetasizer Nano ZS.^20^ Finally, the pH of the undiluted formulations was monitored at room temperatures using an Accumet pH meter and a micro probe (Fisher Scientific, Canada).

## Results and Discussion

**Figure 1:**
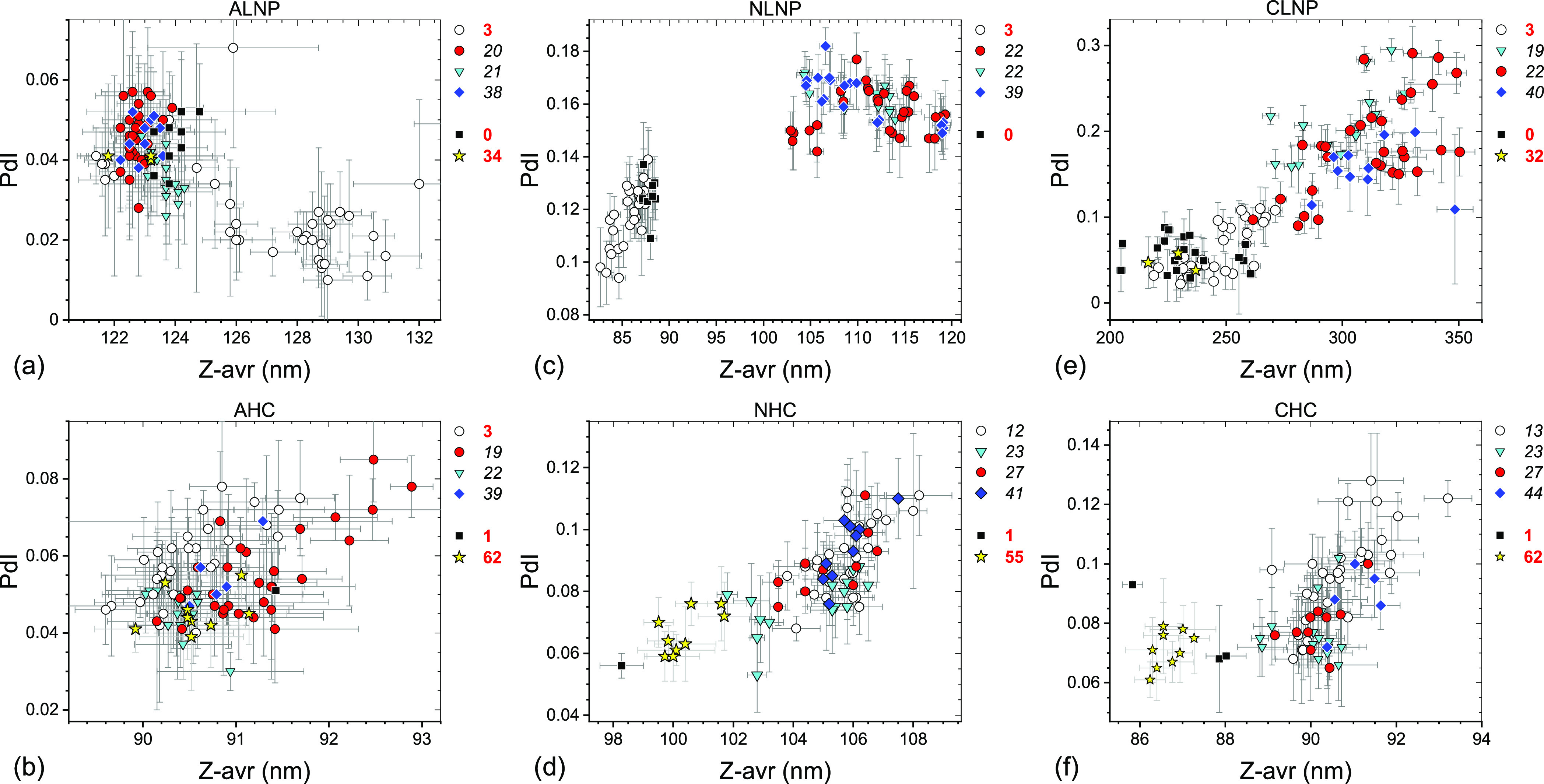
PdI–Z-avr scatter plots of anionic (a,b), neutral (c,d), and cationic (e,f) LNPs (a,c,e) and liposomes (b,d,f) stored for a long term in the −70 °C freezer. Error bars are 1 standard deviation of five repeat measurements. Each point corresponds to a single aliquot. Four data sets are differentiated by symbol shape and color: open circles (set #1), red circles (set #2), cyan triangles (set #3), and blue diamonds (set #4). Approximate storage time in weeks at −70 °C prior to a measurement is indicated next to a set symbol at the top right corner of the graphs. Results of systematic homogeneity analyses for the four top-listed sets in each graph are shown in Table 2. Results of additional reference measurements conducted at INT shortly after the freezing (black squares) or after prolonged storage (yellow stars) are also shown with approximate storage time in weeks indicated to the right of the graphs.

Measurement results of sets #1 through #4 (Table 1) are shown graphically as PdI vs Z-avr scatter plots in Figure 1 and their statistical analysis is summarized in Table 2 (see the Supporting Information for additional details). Also shown in Figure 1 are the results of the reference measurements conducted at INT on a limited number of extra vials stored at −70 °C. What clearly transpires is that there is no statistically significant variation between the set mean Z-avr and PdI values of the LNP sets #2, #3, and #4 and all four liposome sets, which indicates excellent −70 °C storage stability over the corresponding time intervals. In fact, the mean Z-avr values of the LNP sets #2, #3, and #4 vary by approximately 0.7% for ALNP, 1.7% for NLNP, and 4.4% for CLNP and those of all four liposome sets by only as little as 0.9% for AHC, 1.2% for NHC, and 1.0% for CHC. The mean PdI of the same measurement sets vary by 0.02 or less except for CLNP, for which it varies by 0.05. The LNP sets #2–#4 and the liposome sets #1–#4 show no deterioration of overall size homogeneity during the corresponding time intervals even though individual homogeneity indicators, the between-vial (*s*_bb_) and within-vial (*s*_w/in_) standard deviations, show a small variation. Both *s*_bb_ and *s*_w/in_ values are relatively small and, therefore, Z-avr coefficients of variation are as small as ≤0.8% for AHC, ≤1.7% for NHC, ≤1.1% for CHC, and ≤0.5% for ALNP and only somewhat larger for NLNP (≤4.9%) and CLNP (≤7.9%).

**Table 2:** Results of the Homogeneity Analyses of the Six LNP and Liposome Formulations^b^

formulation (set #)	mean Z-avr (nm)	*s*_bb_ (nm)	*s*_w/in_ (nm)	*s*_r_ (nm)	*u*_h_ (nm)	mean PdI	*s*_PdI_
ALNP (1)	127.3	2.90	1.40	0.47	3.25	0.027	0.014
ALNP (2)	122.7	0.29	0.30	0.32	0.53	0.046	0.009
ALNP (3)	123.6	0.16	0.42	0.32	0.55	0.036	0.008
ALNP (4)	123.0	0.26	0.46	0.32	0.62	0.046	0.008
NLNP (1)	85.8	1.46	0.37	0.19	1.52	0.118	0.012
NLNP (2)	112.2	5.20	0.45	0.30	5.23	0.157	0.010
NLNP (3)	110.3	3.81	0.33	0.39	3.84	0.162	0.007
NLNP (4)	110.3	5.43	0.26	0.30	5.44	0.162	0.010
CLNP (1)	245.8	11.07	8.95	0.86	14.26	0.060	0.031
CLNP (2)	311.6	22.11	10.17	2.57	24.47	0.185	0.058
CLNP (3)	298.1	7.76	20.10	1.58	21.60	0.207	0.043
CLNP (4)	310.6	18.16^a^		2.39	18.32	0.156	0.035
AHC (1)	90.5	0.18	0.55	0.19	0.61	0.059	0.012
AHC (2)	91.3	0.27	0.61	0.33	0.74	0.055	0.013
AHC (3)	90.4	0.21	0.12	0.20	0.31	0.046	0.008
**AHC (4)**	90.8	0.30^a^		0.26	0.40	0.055	0.010
NHC (1)	105.8	0.77	0.70	0.25	1.07	0.091	0.012
NHC (2)	105.3	1.24	0.45	0.22	1.34	0.089	0.011
NHC (3)	104.5	1.72	0.32	0.23	1.76	0.076	0.011
NHC (4)	105.8	0.58	0.50	0.26	0.81	0.094	0.012
CHC (1)	90.7	0.29	0.90	0.28	0.99	0.097	0.017
CHC (2)	90.2	0.43	0.76	0.16	0.89	0.080	0.011
CHC (3)	90.1	0.65	0.19	0.24	0.72	0.077	0.011
CHC (4)	91.0	0.55^a^		0.23	0.60	0.088	0.012

a Standard deviation of the set measurements.

b Mean Z-avr is a set mean value of the intensity-weighted hydrodynamic diameter; *s*_bb_, *s*_w/in_, and *s*_r_ are between-vial, within-vial, and repeatability Z-avr standard deviations; *u*_h_ is a combined standard uncertainty, and mean PdI and *s*_PdI_ are a set mean value and a standard deviation of the PdI.

Further examination of Figure 1a,c,e and Table 2 leads to two observations: first, the (Z-avr, PdI) pairs of LNP sets #1 significantly differ from those of the LNP sets #2, #3, and #4 pairs and second, in the case of NLNP and CLNP, they coincide, broadly speaking, with the INT reference measurement pairs (black squares and yellow stars). Even though the ALNP case may be considered inconclusive, the INT reference measurement (Z-avr, PdI) pairs appear to coincide with the sets #2, #3, and #4 pairs. For liposome formulations, the set #1 (Z-avr, PdI) pair populations are statistically not different from those of sets #2, #3, and #4. Also, the INT reference measurement (Z-avr, PdI) pairs collocate with those of all four sets of AHC, but neither the sets of NHC or CHC. It is important to point out that the set #1 measurements for the three LNP formulations as well as AHC were conducted after 3 weeks of −70 °C storage but those for NHC and CHC after 12–13 weeks. Based on the above observations, we concluded that the formulations stored at NRC underwent a modification prior to the set #2 measurements and in the case of NHC and CHC even prior to the set #1 measurements that led to a relatively small mean Z-avr value increase for NHC and CHC but a significantly larger one for NLNP (≤25%) and CNLP (≤20%). The modification was tentatively attributed to an overnight freezer malfunction and an inadvertent storage temperature increase to −35 °C for a period of just a few hours. Subsequent review of unpublished results of size measurements on vials used in the cytotoxicity assessment experiments^17^ confirmed the attribution by narrowing the time range of the formulation-modifying event to 3 days prior to and 4 days after the freezer incident. To differentiate in Figure 1 the measurements on vials unaffected and affected by the freezer malfunction, the corresponding vial storage time was shown by bold red and black italic fonts, respectively.

**Figure 2:**
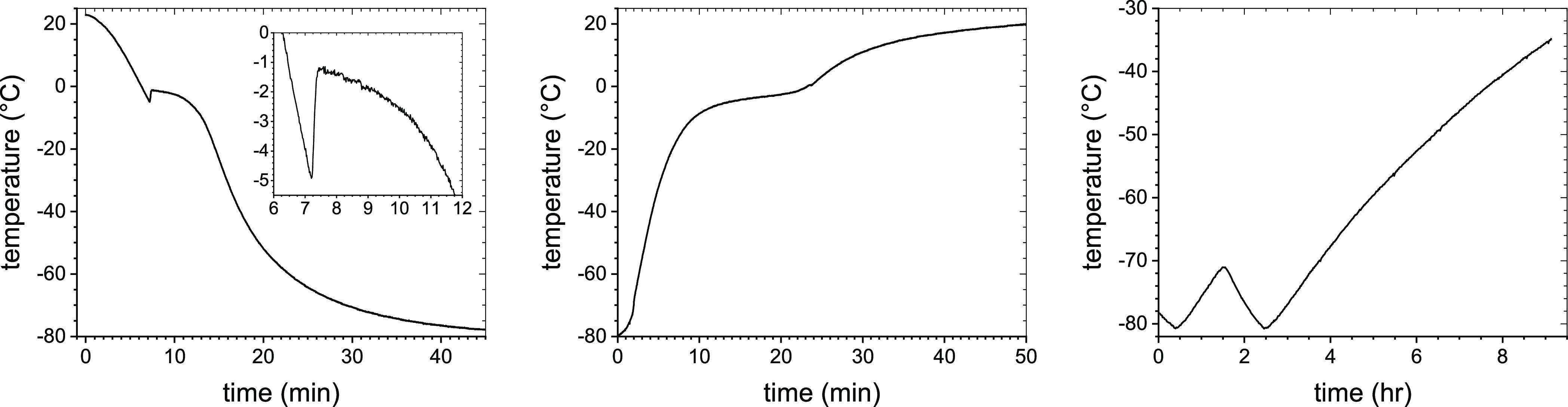
Representative temperature profiles of NLNP during normal freezing (left), NLNP during normal thawing (middle), and the failing freezer interior (right).

To better understand the effects of the freezer malfunction on our formulations, we recreated the freezer failure incident as closely as possible and monitored the temperature variation in test vials. Taking into account our observations as well as past research on freezing solutions of salts and sugars, we deduced a likely sequence of events during the freezer malfunction. Upon freezing of a biopharmaceutical formulation such as LNPs or liposomes dispersed in dPBS/sucrose, it first supercools by as much as several degrees Celsius, with the exact value depending on the cooling rate, before ice nucleation is initiated, and its temperature rapidly increases due to a release of the latent heat to near its depressed equilibrium freezing temperature. We estimated experimentally (see Figure 2) that during the initial freezing, the formulations reported in this work supercooled down to −5 °C before their temperatures rapidly increased up to −1.3 °C, in a good agreement with Blagden law prediction for a 0.5x dPBS/15% sucrose solution. Growth of ice crystals, slow cooling, and eventually freezing of the formulation follow.^21^ Since the freezing water forms nearly pure ice crystals, the concentration of the solution surrounding the crystals, the so-called freeze concentrated solution (FCS), dramatically increases. For example, the concentration of an isotonic NaCl solution increases 20 times when cooled down to −10 °C; other FCS components show a similar concentration increase.^22^ Increasing concentration may lead, among others, to supersaturation of sucrose^22^ and precipitation of salts; disodium phosphate (Na_2_HPO_4_·12H_2_O) is the first to precipitate after the onset of ice crystallization, leading to an abrupt drop of pH. For an 8 mM phosphate solution, which approximately corresponds to the partial phosphate concentration in 1x dPBS, the pH drop was reported to be greater than 2 units for an initially neutral solution (pH = 7.4), resulting in acidification of the microenvironment.^23^ However, the presence of sugars was observed to limit the pH drop.^24^ An opposite and possibly greater pH shift can be caused by trapping of chloride ions within the growing ice crystals. Since chloride ions are known to incorporate into ice more efficiently than Na^+^, a flow of hydronium ions into the ice to compensate for the charge misbalance results in basification of FCS, which in a case of slow freezing or in the presence of small particles in a formulation could lead to an upward pH shift of greater than 5 units.^25^ At lower temperatures below the NaCl eutectic temperature (−21.2 °C), hydrated NaCl precipitates along with ice.^26^ In addition, freezing water can form a variety of ice crystals such as platelets, dendrites, fractals, or needle-like structures,^27^ which may result in a complex ice morphology, with crystals surrounded by or encapsulating pockets of FCS. Consequently, due to highly heterogeneous morphology and chemical composition, the microenvironment properties strongly vary across the system with, among others, large point-to-point pH variation^28^ and mechanical, electrical, or chemical stress at the ice/FCS interface.^29^ In effect, depending on the length of time the formulation spends in intermediate states between the liquid dispersion and a frozen solid, growth, aggregation, disintegration, or morphological alteration of the nanoparticles can take place.

Our test measurements show (see Figure 2) that it took approximately 1 min to supercool the formulation from 0 °C down to −5 °C, (10 to 15) s to warm up to −1.3 °C, and over 20 min to cool down to −70 °C (with the freezer temperature at −80 °C). Thawing the vials at ambient conditions prior to the measurements took approximately (20 to 25) min for their temperature to rise from −80 °C to 0 °C and another (20 to 25) min for the formulations to reach room temperature. Thus, our nanoparticles spent up to 45 min in total at temperatures between −70 °C and 0 °C during the initial freezing and the pre-measurement thawing, with, however, only a small fraction of the time in a strongly varying pH environment above the FCS glass-transition temperature, *T*_g_’ (see below). While we have not measured the glass-transition temperature of FCS in our formulations, we determined *T*_g_’ for the 1x dPBS/30% sucrose solution as approximately equal to (−45 to −43) °C, which is consistent with previously obtained values for NaCl/sucrose solutions.^30^ Since *T*_g_’ depends on the solution composition but not the initial concentration,^22^ its value should not be much different in our formulations. On the other hand, during the freezer malfunction that resulted in a slow and steady temperature increase up to −35 °C, the formulations were exposed to temperatures above −70 °C for a minimum of 6.5 h, of which, for a minimum of 2 h, the formulations were exposed to temperatures above *T*_g_’ when our nanoparticles resided in a strongly heterogeneous environment, with the pH widely differing from the nominal room temperature value of 7.0 to 7.1.^22^ The increasing pH of FCS, likely over the isoelectric point of the neutral and cationic LNPs and liposomes, resulted in a transitory neutralization of their surface charges and, consequently, Z-avr increase for NLNP, CLNP, NHC, and CHC. As for the anionic LNPs and liposomes, the exposure to the highly basic environment even further stabilized the formulations. In effect, the ALNP mean Z-avr value slightly decreased from that of the ALNP set #1. For AHC, both Z-avr and PdI remained unchanged.

It has previously been claimed that subjecting LNP formulations to repeated freeze–thaw cycles may result in the growth of nanoparticles due to stress exerted on LNPs during crystallization.^15^ It remains not entirely clear to us whether the mean Z-avr size increase for neutral and cationic LNPs and liposomes was the result of the growth of primary particles driven by a molecular-level mechanism such as hydration or osmotic forces or a net effect of an onset of LNP aggregation.^31,32^ This problem is being addressed by additional experiments and will be discussed in a future publication. It should also be pointed out that the detection of the size and size distribution change due to the temporary storage temperature increase up to approximately −35 °C was only possible due to the very low initial polydispersity of our formulations, low between-vials and within-vial heterogeneity, as well as much larger test set sizes (*n*) and higher measurement precision as compared with LNP stability analyses previously reported by other groups.

**Figure 3:**
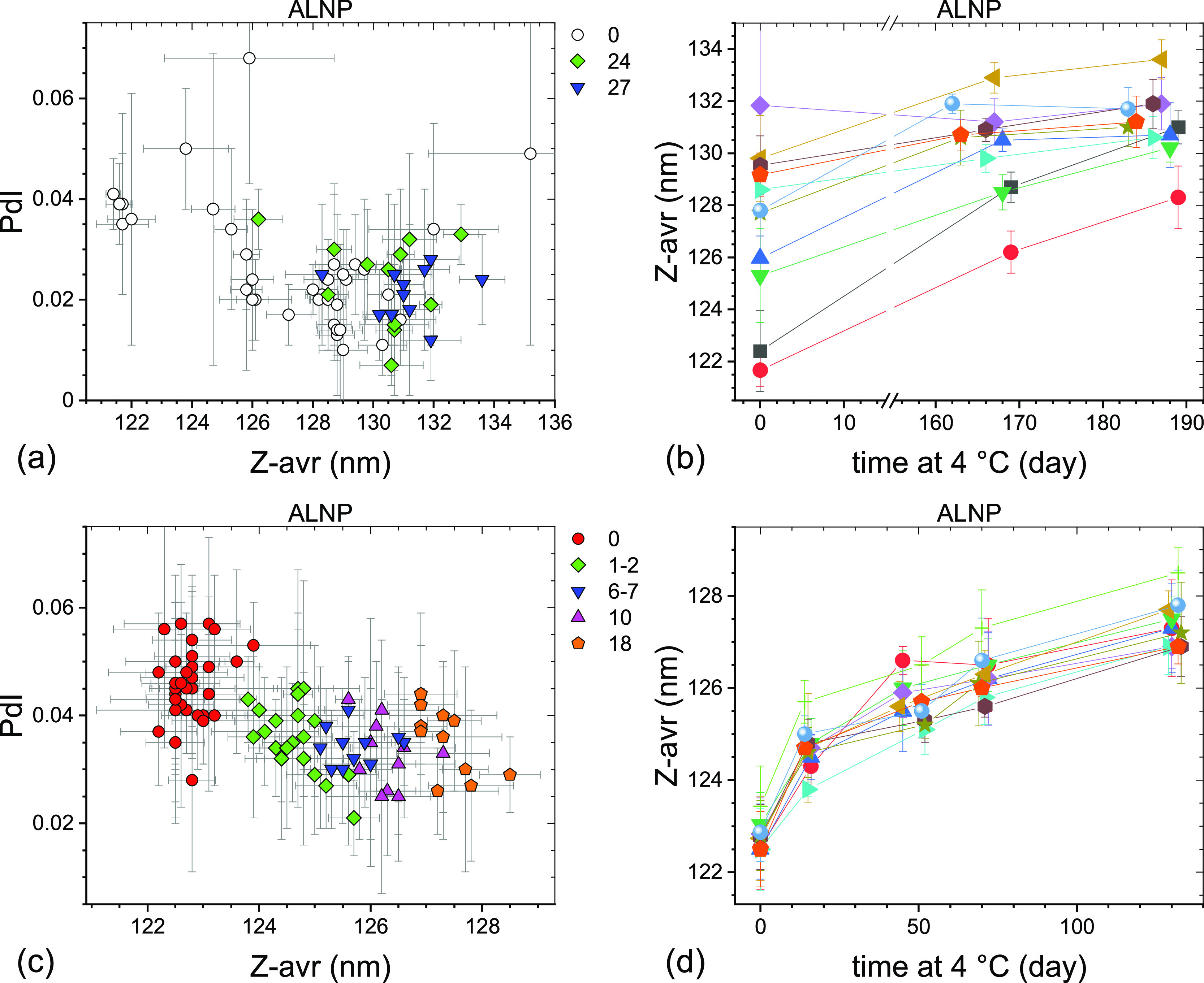
PdI–Z-avr (a,c) and Z-avr–time (b,d) plots illustrating the size and polydispersity evolution of two sets of anionic LNPs stored at 4 °C. The sets #1 (a,b) and #2 (c,d) were initially stored in a −70 °C freezer for 3 and 20 weeks, respectively. Each point corresponds to a single aliquot except for the day 0 points in (b,d) where average values of three aliquots per vial are shown. Error bars are 1 standard deviation of five repeat measurements. Data sets corresponding to approximately the same storage duration are shown in (a,c) with the same symbol shape and color. The approximate storage duration in weeks at 4 °C prior to a measurement is indicated in (a,c). Data points in (b,d) corresponding to the same vial are connected by guide lines and shown with the same symbol shape and color.

**Figure 4:**
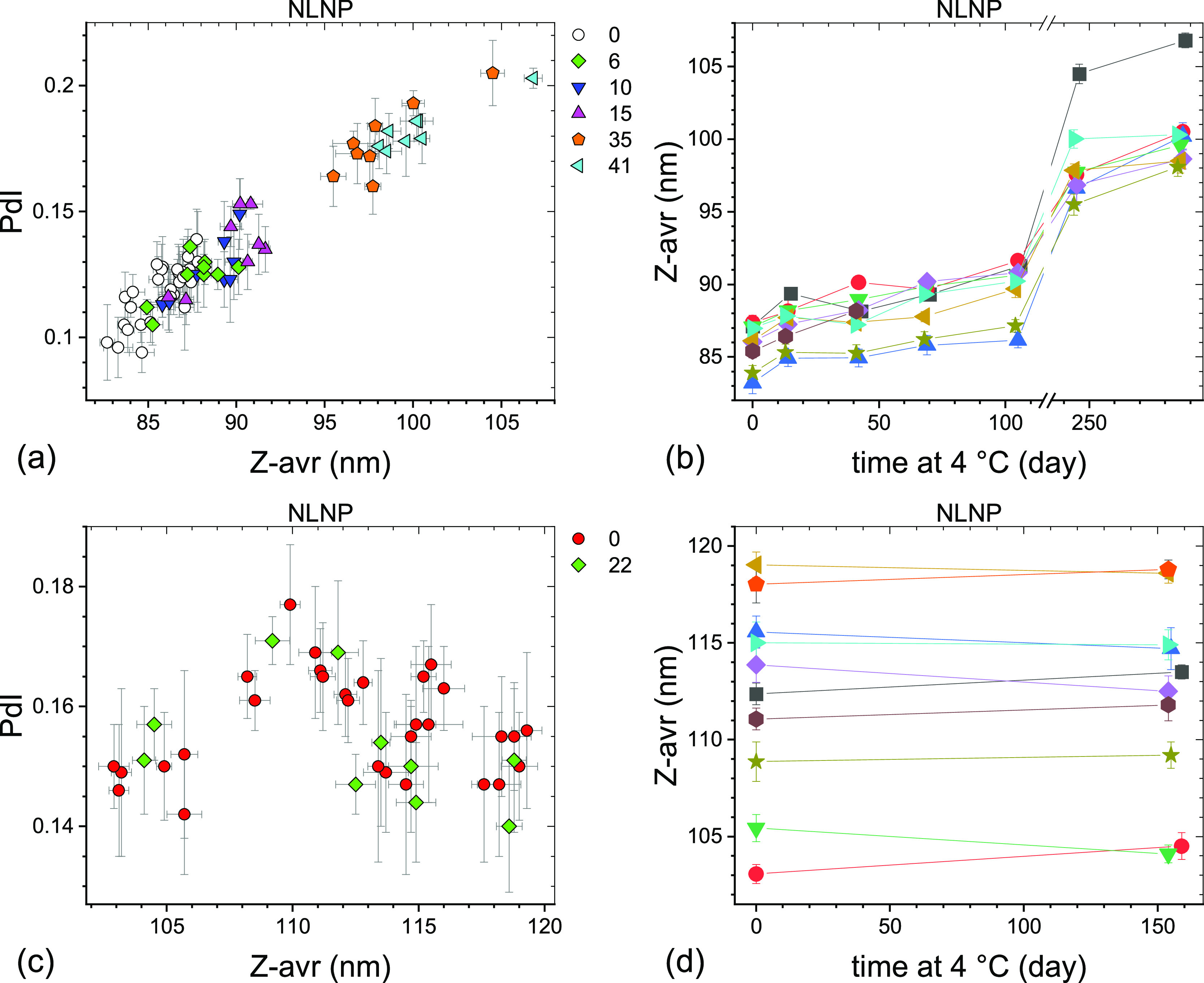
PdI–Z-avr (a,c) and Z-avr–time (b,d) plots illustrating the size and polydispersity evolution of two sets of neutral LNPs stored at 4 °C. The sets #1 (a,b) and #2 (c,d) were initially stored in the −70 °C freezer for 3 and 22 weeks, respectively. Each point corresponds to a single aliquot except for the day 0 points in (b,d) where average values of three aliquots per vial are shown. Error bars are 1 standard deviation of five repeat measurements. Data sets corresponding to approximately the same storage duration are shown in (a,c) with the same symbol shape and color. The approximate storage duration in weeks at 4 °C prior to a measurement is indicated in (a,c). Day 7 and 8 points shown in (b) are omitted in (a) for clarity. Data points in (b,d) corresponding to the same vial are connected by guide lines and shown with the same symbol shape and color.

**Figure 5:**
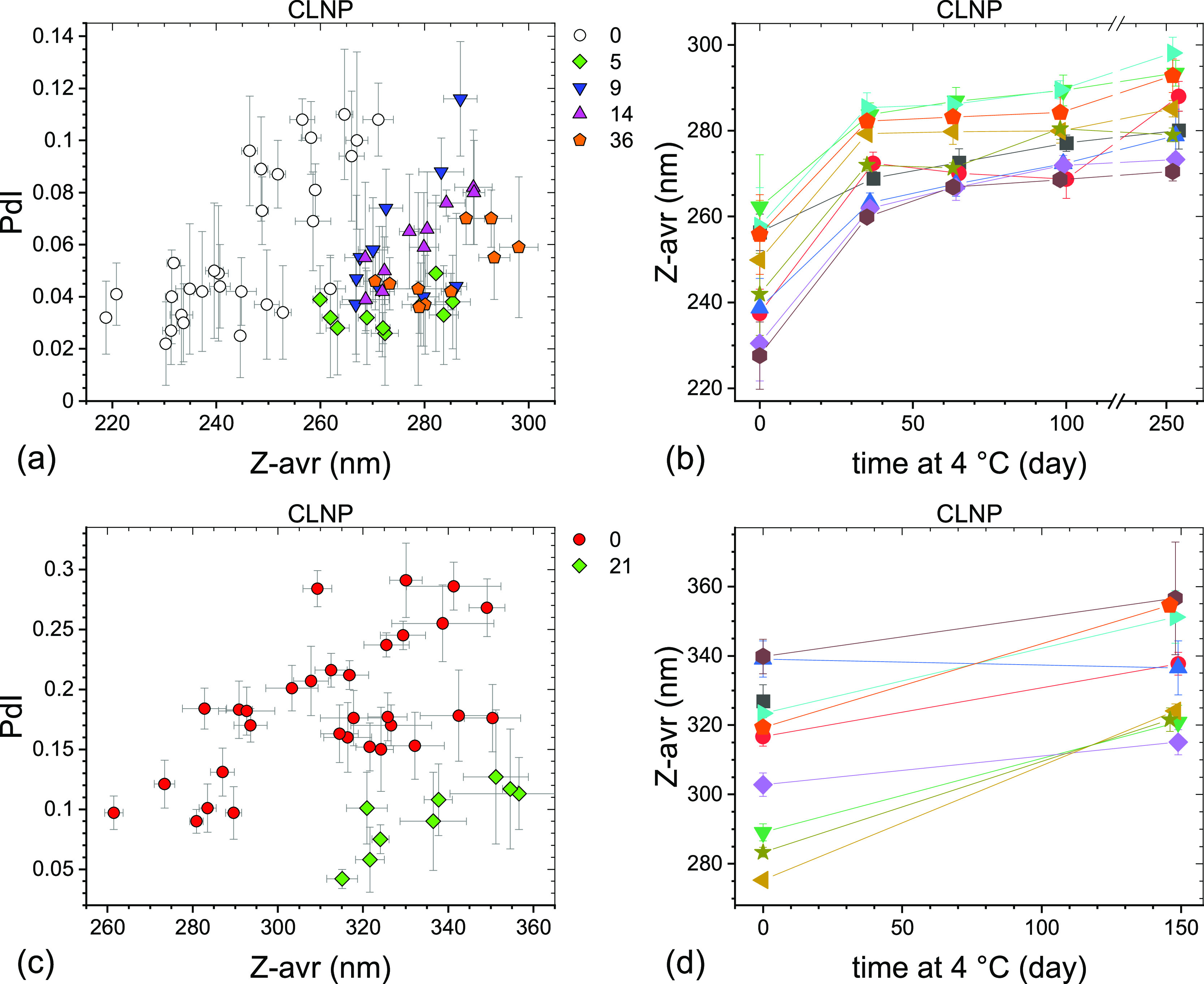
PdI–Z-avr (a,c) and Z-avr–time (b,d) plots illustrating the size and polydispersity evolution of two sets of cationic LNPs stored at 4 °C. The sets #1 (a,b) and #2 (c,d) were initially stored in the −70 °C freezer for 3 and 22 weeks, respectively. Each point corresponds to a single aliquot except for the day 0 points in (b,d) where average values of three aliquots per vial are shown. Error bars are 1 standard deviation of five repeat measurements. Data sets corresponding to approximately the same storage duration are shown with the same symbol shape and color. The approximate storage duration in weeks at 4 °C prior to a measurement is indicated in (a,c). Data points in (b,d) corresponding to the same vial are connected by guide lines and shown with the same symbol shape and color.

**Figure 6:**
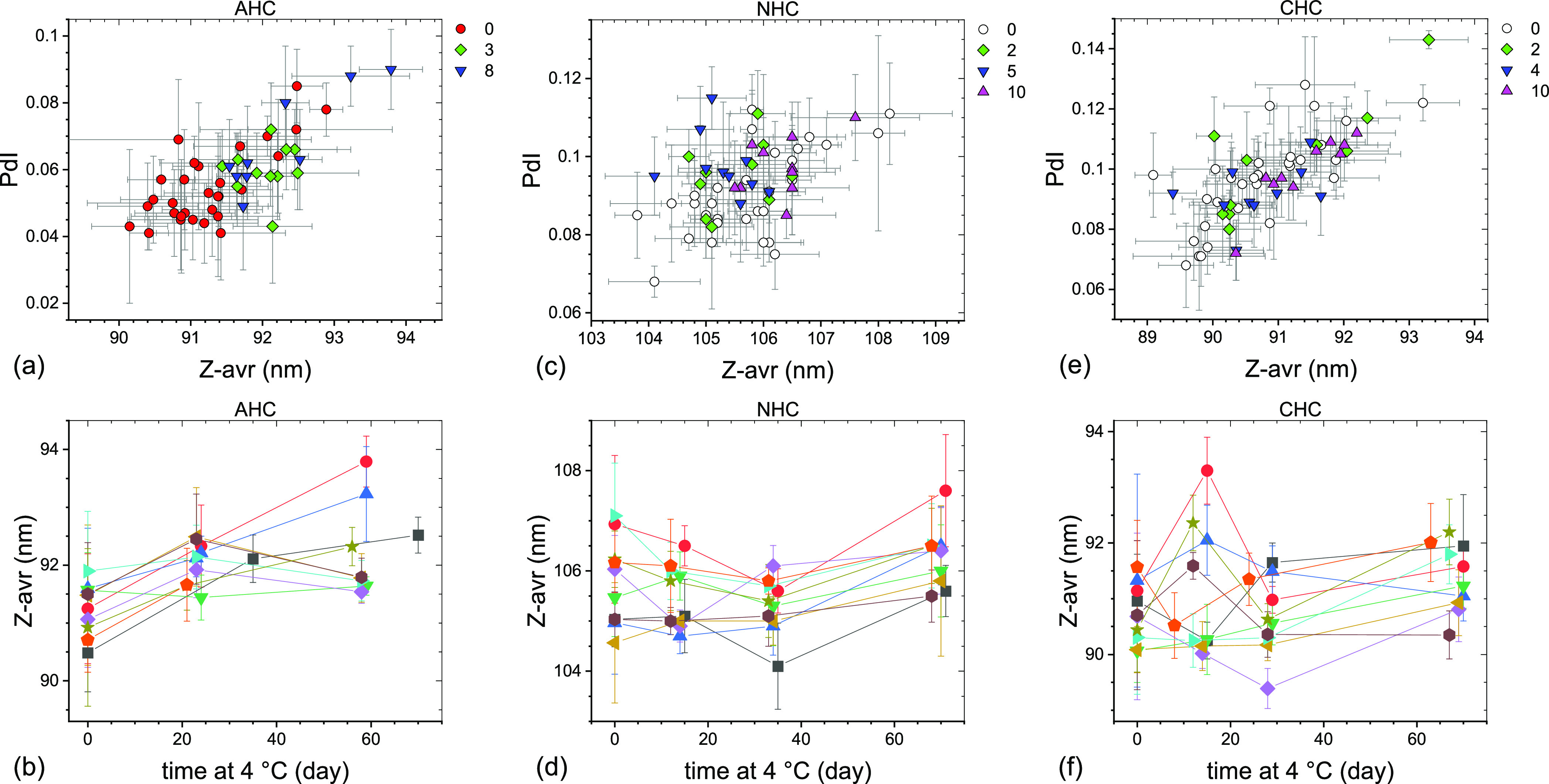
PdI–Z-avr (a,c,e) and Z-avr–time (b,d,f) plots illustrating the size and polydispersity evolution of anionic (a,b), neutral (c,d), and cationic (e,f) liposomes stored at 4 °C. Each point corresponds to a single aliquot except for the day 0 points in (b), (d,f) where average values of three aliquots per vial are shown. Error bars are 1 standard deviation of five repeat measurements. Data sets corresponding to approximately the same storage duration are shown in (a,c,e) with the same symbol shape and color. The approximate storage duration in weeks at 4 °C prior to a measurement is indicated in (a,c,e). Data points in (b,d,f) corresponding to the same vial are connected by guide lines and shown with the same symbol shape and color.

Particle size and size distribution of the LNP formulations stored at 4 °C were monitored for the sets #1 and #2 over varying periods of time up to over 40 weeks. The results are presented graphically for ALNP in Figure 3, NLNP in Figure 4, and CLNP in Figure 5. The Z-avr diameter of LNPs generally increased with storage time, but the degree of change varied between formulations, vial sets, as well as time points and the overall size change was small. PdI values of LNP generally decreased with time or remained unchanged except for the NLNP set #1 for which a systematic increase was observed (Figure 4a). The initial between-vials size heterogeneity, as indicated by the spread of the Z-avr values, appeared to somewhat decrease during the first few months of storage at 4 °C for ALNP set #1 (Figure 3b), NLNP set #2 (Figure 4d), and CLNP sets #1 and #2 (Figure 5b,d) and remain unchanged for the ALNP set #2 (Figure 3d) and NLNP set #1 (Figure 4b). The particle size evolution for ALNP set #2 (Figure 3c,d), NLNP set #1 (Figure 4a,b), and CLNP set #1 (Figure 5a,b) was monitored more frequently than for the other sets over the initial 2 months of storage at 4 °C and we observed that, on average, Z-avr increased fastest over the initial 2–3 weeks with a slower size change over the following months. In contrast to LNP, no statistically significant change of mean Z-avr and PdI was observed for the liposome formulations over the initial 2–3 months of storage at 4 °C, as shown graphically in Figure 6.

A similar pattern of LNP size increase with time to that observed in the present work has previously been reported by other groups and attributed to Ostwald ripening, for example, in the work by Gindy et al.^33^ or by Suzuki et al.^34^ Gindy et al.^33^ demonstrated that through rational optimization of LNP physical and macromolecular properties, such as LNP size or macromolecular packing, the Ostwald ripening instability could be significantly attenuated or entirely eliminated. However, it is important to point out that the rates of LNP growth in refs (33) and (34) are significantly larger than those observed in our work. In addition, the size evolution of our LNP stored at 4 °C does not appear to follow the classical Lifshitz–Slyozov–Wagner theory of Ostwald ripening.^35^ While the exact identity of a molecular-level process responsible for the relatively small size increase with time of our LNP is presently unknown, the process has to be consistent with the following three observations.

**Figure 7:**
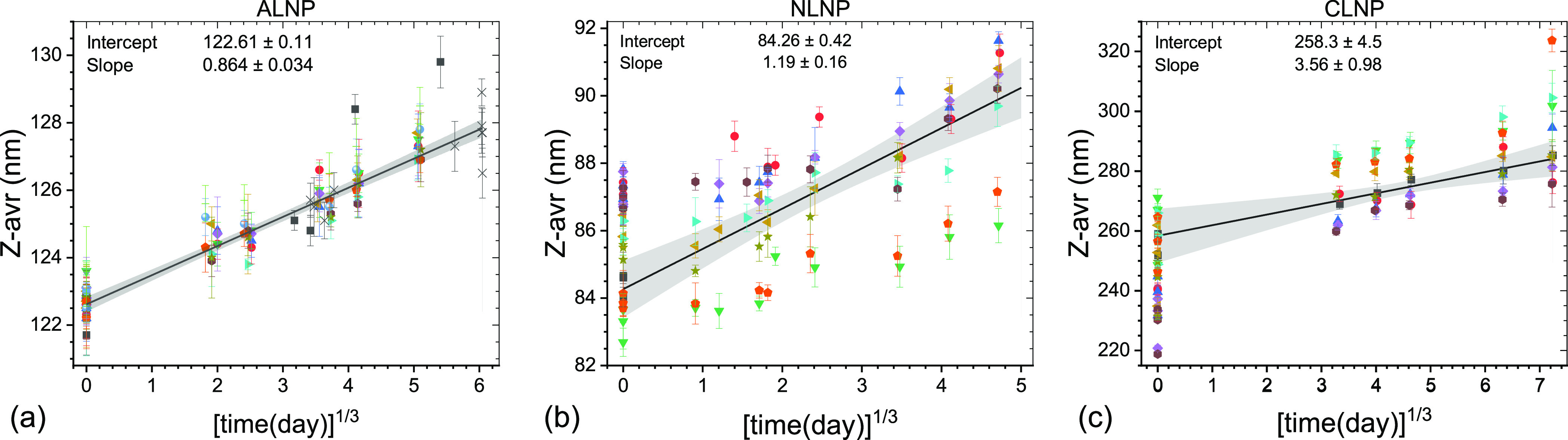
Z-avr vs cube root of time plots for selected LNP sets illustrating ripening of LNP during storage at 4 °C: (a) ALNP set #2, (b) NLNP set #1, and (c) CLNP set #1. The best fit lines and the confidence intervals are shown by black solid lines and grayed areas.

First, even though the growth rates differ between the LNPs, the particle size increase appears to follow the ⟨Z-avr⟩ ∼ *t*^1/3^ + const dependence for at least the first 5–6 months of storage at 4 °C, where ⟨Z-avr⟩ is the mean Z-avr value and *t* is the storage time. Figure 7 shows ⟨Z-avr⟩ ∼ *t*^1/3^ fits for three LNP data sets. The average rates of Z-avr growth with *t*^1/3^ range from 0.864(34) nm/day^1/3^ for ALNP set #2, to 1.19(16) nm/day^1/3^ for NLNP set #1, to 3.56(98) nm/day^1/3^ for CLNP set #1. However, it is interesting to note that the relative rates calculated as the slope-to-intercept ratios are the same for NLNP set #1 and CLNP set #1 at 0.014 day^–1/3^, while the one for ALNP set #2 is half as small at 0.007 day^–1/3^. In contrast to ALNP, the NLNP and CLNP fits do not include the initial (day zero) points as the growth appeared to be hindered over the initial few hours to half a day in the case of NLNP and to proceed initially faster in the case of some of the CLNP vials.

Second, the LNP size increase with time is accompanied by narrowing of the size distribution, as indicated by decreasing PdI values (see Figures 3a,c, 4c, and 5a,c). NLNP set #1 (Figure 4a) may be a more complex case as PdI does not change much over the initial 4 months or so, but the later data points show a significant increase of both PdI and Z-avr values. An additional indication of narrowing of size distributions is the decreasing with time of between-vials size heterogeneity, as discussed above (see, e.g., Figures 3b or 5d). Fitting an evolution of each vial separately rather than in a global fit (Figure 7b, NLNP set #1) shows that the growth rates vary widely between vials, (0.54 to 1.34) nm/day^1/3^, and appear to inversely correlate with Z-avr values (smaller particles growing faster). Two vials coded by orange circles and green triangles in Figure 7b appear not to follow the same trend as the other seven vials, with the growth rates similar to the seven-vial average, but the initial (intercept) Z-avr values some 3 nm smaller.

Third, the LNP growth appears to preserve the shape of the initial Z-avr distributions, as illustrated indirectly for the large fraction of vials by the approximately parallel or slightly convergent segments of the guide lines connecting same vial points [Figures 3b,d, 4b,d and 5b,d].

The three observations discussed above are characteristic of a process in which the overall volume of particles grows linearly with time at a constant particle number density.^36^ The growth can be triggered by a thermal drift or any other activation mechanism that results in a sustained flux of molecules at a sufficiently small feeding rate that only affects the level of supersaturation but not the diffusion. We hypothesize that the growth of LNP, but not liposome particles, is triggered by a small imbalance of a sucrose equilibrium in a freshly thawed LNP formulation. It has been shown that sugar molecules hydrogen bond with lipid head groups replacing water molecules.^37,38^ It has also been demonstrated that in the case of liposomes, addition of sugars leads to their size increase.^39^ While both LNP and liposome frozen formulations contain a significant amount of sucrose, 15% (w/v), the LNP formulations were initially prepared without sucrose, which was only added shortly before freezing to produce the final formulations of LNP in 0.5x dPBS/15% sucrose. Therefore, it is reasonable to assume that a medium/nanoparticle sucrose equilibrium might not had been attained prior to the freezing, thus triggering a slow LNP growth during storage at 4 °C. Since, on the other hand, the liposome formulations were produced starting with outright dissolution of lipid films in 0.5x dPBS/15% sucrose, no sucrose imbalance was present upon thawing and therefore, no particle size growth during storage at 4 °C was observed for them. We should point out that an incomplete salt equilibration prior to the initial freezing due to the change of the dPBS concentration from 1x to 0.5x following the sucrose addition could have triggered the growth of LNP particles at 4 °C as well.^31,32^ One might also consider the inadvertent exposure of the formulations to the temperatures above the glass-transition and below the eutectic temperatures during the freezer malfunction to be responsible for the particle growth during storage at 4 °C. However, this alternative is inconsistent with two observations: (1) both LNP sets #1 and #2 showed a 4 °C size increase (Figures 3, 4, and 5), but only the set #2 was affected by the freezer malfunction and (2) the liposome formulations showed essentially no particle size change at 4 °C (Figure 6) even though the AHC set #2 and NHC and CHC sets #1 and #2 were affected by the freezer malfunction. Systematic investigation, opposite to the exploratory analyzes presented in this work, will be required to understand the triggering mechanism of the 4 °C LNP size growth process. Partial results for formulations stored at 4 °C for periods of time longer than 6 months indicate that the growth of LNP particles may accelerate for some of the formulations presumably due to another process, gaining significance at longer storage times (see time >250 days points in Figures 4b and 5b).

## Conclusions

Six LNP and liposome formulation candidate reference materials of varying particle surface charge and size were produced (200 vials each) and analyzed for size, size distribution, size homogeneity, and long-term storage stability. To the best of our knowledge, our work is the first attempt to develop a reference material of LNPs. It is also the most extensive and most precise characterization of LNP size and size homogeneity and stability attempted so far. The formulations were stored for a long term at −70 °C and remained stable with respect to size and size distribution, as determined by the Z-avr hydrodynamic diameter and PdI polydispersity measurements, for a minimum of 9 months despite an inadvertent short-time temperature elevation up to −35 °C due to a freezer malfunction. All six formulations analyzed in this work were highly monodisperse as produced with the initial mean PdI values ranging from 0.027 for ALNP to 0.118 for NLNP, and they remained monodisperse following the freezer failure incident. For four out of six formulations, the mean PdI values remained <0.1, with a factor of 3 smaller than frequently observed for optimized drug delivery LNP formulations (see, e.g., refs (8, 40, and 41)), and even for the most affected CLNP formulation, the mean PdI value did not exceed 0.2 (see Figure 1 and Table 2). With respect to the mean particle size, the freezer incident did not affect the anionic liposomes but resulted in a small (≤5%) increase of the mean Z-avr diameter of the neutral and cationic ones. A larger size increase (≤25%) was observed for NLNP and CLNP, but the ALNP mean Z-avr value slightly decreased. The LNP formulations stored at 4 °C for several months showed a small particle Z-avr size increase proportional to the cube root of the storage time. The size increase appeared to be accompanied by narrowing of the size distribution, as observed for several of the measurement sets. The small particle size and size distribution evolution of the LNP formulations stored at 4 °C were attributed to a small sucrose imbalance resulting from a pre-freeze sucrose addition and dilution of the stock formulations. No size or polydispersity change was observed for liposome formulations stored at 4 °C for up to 3 months. Detection and evaluation of the size and size distribution changes, both those resulting from the freezer incident and those observed during the 4 °C storage, were largely possible due to the high monodispersity of the formulations, high between-vials and within-vial homogeneity, large size of the analysis sets (up to 10 or more vials per set and 3 aliquots per vial), and high precision of the measurements.

## Supporting Information Available

Extended description of characterization methods and data analysis (PDF)
