# Supplementary material for: Lipid Nanoparticle and Liposome Reference Materials: Assessment of Size Homogeneity and Long-Term −70 °C and 4 °C Storage Stability
Source: Langmuir. 2023 Feb 7;39(7):2509–19. doi: 10.1021/acs.langmuir.2c02657 (PMC9948293; doi:10.1021/acs.langmuir.2c02657)
Supplement: Supplementary file 1 — la2c02657_si_001.pdf [file la2c02657_si_001.pdf]

Supporting Information

Lipid nanoparticle and liposome reference materials: assessment of size homogeneity and long-term −70 °C and 4 °C storage stability

Zygmunt J. Jakubek[a,], Sam Chen[b], Josh Zaifman[b], Yuen Yi C. Tam[b], and Shan Zou[a]*

[a] Metrology Research Center, National Research Council Canada, Ottawa, Ontario K1A 0R6, Canada; zygmunt.jakubek@nrc.ca (Z.J.J); shan.zou@nrc.ca (S.Z.)

[b] Integrated Nanotherapeutics Inc., 205-4475 Wayburne Drive, Burnaby, British Columbia V5G 4X4, Canada; samchen@integratedntx.com (S.C.); jzaifman@integratedntx.com (J.Z.); christam@integratedntx.com (Y.Y.C.T.)

Particle Size Characterization	S1
Long Term Storage Stability and Homogeneity Assessment	S2
Additional Characterization	S3
Analysis and Results	S3

Particle Size Characterization. Mean particle size and particle size distribution of the LNP and liposome formulations were evaluated by DLS using Zetasizer Nano ZS (Malvern Instruments Ltd., Worcestershire, UK). In advance of a measurement, selected vials and diluents were equilibrated at ambient lab temperatures. Measurement samples were prepared by diluting 10 µL aliquots of each formulation 50 folds in 1x dPBS for the target concentration of 60 µg/mL for NLNP and 40 µg/mL for the other five formulations. The dilutions were prepared directly in an analysis cuvette (ZEN0040, Malvern Panalytical, UK or Fisherbrand Polystyrene Semi-micro, Fisher Scientific, Canada) and thoroughly homogenized by repeated pipetting, taking care to avoid injection of air bubbles. After insertion into the sample compartment, samples were equilibrated at the measurement temperature (25.0±0.1) °C for minimum 3 minutes with extra equilibration time applied whenever the room temperature was lower than 21.5 °C. Five repeat indications were acquired for each measurement with each indication consisting of twenty-one 10-s-long runs.

The measurements were processed and analyzed with the Zetasizer Software (ver. 7.11 or ver. 8.0, research grade; Malvern Panalytical, UK). Default values for all analysis parameters except for those listed in Table S1 below were used. Z-average (Z-avr) hydrodynamic diameter (sphere-equivalent scattered light intensity-weighted harmonic average hydrodynamic diameter) and polydispersity index (PdI) were the primary size and size distribution measurands used for

* zygmunt.jakubek@nrc.ca

characterization of the six formulations. Z-avr and PdI are determined by cumulants analysis that is a robust method applicable to dynamic light scattering on monomodal and nearly monodisperse particle dispersions [1]. It is a convenient method for monitoring stability of nearly monodisperse systems such as the six formulations studied in this work since it can efficiently detect systematic nanometer-range mean particle size variation and is sensitive to an onset of aggregation. In analyses using default settings implemented in the Zetasizer Software, the long correlation-time section of the correlation function with values below 10% of the intercept is excluded from the analysis, which assures that the baseline noise or presence of dust particles has no or a minimal effect on the Z-avr value of the sample. While isolated aggregates or agglomerates may also avoid detection due to the truncation as well as quadratic weighing of the correlation function data points, any meaningful level of particle aggregation can be detected by monitoring Z-avr and PdI trends and examination of goodness of fit indicators. It should however be pointed out that it is not always straightforward nor even possible to distinguish between an onset of agglomeration and, for example, swelling of particles based exclusively on observation of the change of Z-avr values, particularly when extent and precision of the measurements is limited; in such cases advance analysis of particle size distribution by other DLS methods such as the non-negative least squares method or other techniques may be required.

Table S1. Relevant effective parameters used in processing of dynamic light scattering measurements at 25 °C.

Sample	Dispersion medium	Viscosity, η (mPa s)	Dielectric constant, ε_r	Refractive index, n_w
Size	0.99x dPBS, 0.3% sucrose	0.9112		1.334
Zeta potential[1]	0.05 dPBS, 1.5% sucrose	0.9270	77.8	1.334

[1] Smoluchowski approximation value 1.5 was used for the Henry function, f(κa). More accurate values determined by Ohshima approximation using measured particle sizes range 1.27–1.32.

Long Term Storage Stability and Homogeneity Assessment. LNP and liposome candidate reference material formulations were stored long term at a −70 °C (nominal temperature) freezer. The actual storage temperature typically ranged −80 °C to −70 °C. For air shipment between laboratories, the formulations were packed inside an insulated dry ice box. Storage stability and homogeneity of the formulations were monitored by periodically analyzing size and size distribution (Z-avr and PdI) of dispersed particles. In advance of a DLS measurement, selected vials were removed from the −70 °C freezer and left at ambient temperatures, normally (21.5 to 23.0) °C, for (40 to 60) minutes to thaw and thermally equilibrate. Four sets of vials drawn by random stratified selection from each 200-unit batch of the −70 °C-stored formulations were analyzed following either the (approximately) balanced nested design (larger sets) or the basic design (smaller sets), as described in the ISO Guide 35 [2]. Three sets were analyzed at NRC and one at INT. Ten vials for each formulation with three aliquots per vial were analyzed for the sets #1 and #2 (with a few exceptions: additional vials measured for ALNP and only 5 vials for NHC and CHC in the set #2). The set #3 measurements were conducted at INT using five vials for each formulation and three aliquots per vial; the vials were long-term stored at NRC and airmailed back to INT prior to the measurements. Minimum five vials with one or two aliquots per vial were analyzed for the sets #4. The original protocol called for 10 vials with 3 aliquots each for the set #1 and set #2 measurements, 5 vials with 3 aliquots each for the set #3 measurements, and 5 vials

with 2 aliquots each for subsequent measurements. The sets #1 were to be measured within one month following the production of the formulations, the sets #2 and #3 both after 4 to 5 months, and the subsequent measurements periodically every 4 to 5 months. The protocol was generally followed for the sets #1, #2, and #3 measurements, but the schedule could not be maintained for the set #4 and the subsequent (not reported here) measurements for a variety of reasons such as a freezer incident, limited resources, coordination, etc. Additional size and size distribution measurements were conducted on a small number of vials originally retained for reference and stored at INT at −70 °C. Upon completion of the measurements, the vials were transferred to a 4 °C refrigerator (3 °C to 5 °C normal temperature range) for long-term storage. For the 4 °C storage stability assessment, two sets of vials, the sets #1 and #2, were periodically removed from a refrigerator for analysis, equilibrated at ambient temperatures for minimum 0.5 hr, and placed back in the refrigerator for further storage after completion of the DLS measurements. The original 4 °C storage stability monitoring protocol called for periodic measurements of all set #1 and set #2 vials. However, limited resources, exhaustion of some of the vials for other characterization, and other factors necessitated disruption of the initially designed measurement schedule and modification of the data point structure.

Additional Characterization. For selected representative vials electrophoretic mobility and zeta potential were measured with Zetasizer Nano ZS by mixed mode measurement phase analysis light scattering (M3-PALS) [3]. Aliquots of 100 µL were diluted tenfold in water for injection (WFI) (HyClone, Fisher Scientific, Canada) to obtain the target concentration of 300 µg/mL for NLNP and 200 µg/mL for the other formulations. All dilutions were prepared in a disposable 4 mL polystyrene cuvette and subsequently loaded into a folded capillary cuvette (DTS1070, Malvern Panalytical, UK) with a 1 mL syringe. Measurements were conducted at (25.0±0.1) °C and 50 V applied voltage. Following minimum 3 minute-long sample equilibration in the sample compartment, five repeat indications separated by a 30 s pause were recorded for each measurement with minimum 10 and maximum 100 runs for each indication. The measurements were controlled and raw data processed and analyzed with the Zetasizer Software using default parameters except for those listed in Table S1. Finally, pH of undiluted (and thawed) formulations was monitored using an Accumet pH meter and a micro probe (Fisher Scientific, Canada).

Analysis and Results. The measurements were analyzed using one-way ANOVA, except for the set #4 of CLNP, AHC, and CHC, for which only one aliquot per vial was measured. For each set, mean Z-avr and PdI values were determined and size homogeneity assessed by calculating Z-avr within vial, between vials, and repeatability standard deviations. No statistically significant trends as a function of the vial order were observed for Z-avr and PdI values of the sets #1 and #2 of any of the six formulations; no vial order trends were tested for the remaining sets.

Z-avr coefficients of variation of the LNP sets #2–#4 and the liposome sets #1–#4 were calculated as relative standard uncertainties of a single Z-avr measurement (consisting of five indications), u_h/Z-avr, where $u_h = (s_{bb}^2+s_{w/in}^2+s_r^2)^{1/2}$ is a combined standard uncertainty, s_{bb} and $s_{w/in}$ are the between vials and within vial standard deviations, and s_r is a repeatability standard deviation. Only characterization and homogeneity contributions are included in u_h; other contributions such as short-term and long-term stability must also be considered to assign a combined standard uncertainty of the particle size reference value.

---

1 International Organization for Standardization. *Particle size analysis — dynamic light scattering (DLS)*; ISO 22412:2017; Switzerland, 2017. [https://www.iso.org/standard/65410.html]

2 International Organization for Standardization. *Reference materials — Guidance for characterization and assessment of homogeneity and stability*; ISO Guide 35:2017; Switzerland, 2017. [https://www.iso.org/standard/60281.html]
3 Connah, M.T.; Kaszuba, M.; Morfesis, A. High Resolution Zeta Potential Measurements: Analysis of Multi-component Mixtures. *J. Dispers. Sci. Technol.* **2002**, *23*, 663–669.
